# Unraveling the Central Role of Global Regulator PprI in *Deinococcus radiodurans* Through Label-Free Quantitative Proteomics

**DOI:** 10.3390/proteomes13020019

**Published:** 2025-05-23

**Authors:** Siyu Zhu, Feng Liu, Hao Wang, Yongqian Zhang

**Affiliations:** School of Medical Technology, Beijing Institute of Technology, No. 5 Zhongguancun South Street, Beijing 100081, China; 1120222466@bit.edu.cn (S.Z.); 3220245354@bit.edu.cn (F.L.); 3120211996@bit.edu.cn (H.W.)

**Keywords:** radiation response metalloprotease, label-free quantitative proteomics, DNA damage repair, antioxidant defense, metabolic regulation

## Abstract

Background: *Deinococcus radiodurans*, renowned for its exceptional resistance to radiation, provides a robust model for elucidating cellular stress responses and DNA repair mechanisms. Previous studies have established PprI as a key regulator contributing to radiation resistance through its involvement in DNA damage repair pathways, oxidative stress response, and metabolic regulation. Methods: Building upon these foundations, our study employs label-free quantitative (LFQ) proteomics coupled with high-resolution mass spectrometry to systematically map *pprI* deletion protein networks by comparing the global proteomic profiles of *pprI* knockout and wild-type *D. radiodurans* strains. Results: Under stringent screening criteria, we identified 719 significantly higher and 281 significantly lower abundant proteins in the knockout strain compared to wild-type strains. Functional analysis revealed that PprI deficiency disrupts homologous recombination (HR) repair, activates nucleotide excision repair (NER) and base excision repair (BER) as a compensatory mechanism, and impairs Mn/Fe homeostasis and carotenoid biosynthesis, leading to increased oxidative stress. Furthermore, PprI deficiency induces significant metabolic reprogramming, including impaired purine synthesis, compromised cell wall integrity, etc. Conclusions: These proteomic findings delineate the extensive regulatory network influenced by PprI, revealing coordinated perturbations across multiple stress response systems when PprI is absent.

## 1. Introduction

Radiation, a pervasive environmental threat, inflicts severe biological damage by inducing DNA double-strand breaks, protein oxidation, and the overproduction of reactive oxygen species (ROS) [1,2,3,4,5]. *Deinococcus radiodurans*, a microorganism renowned for its extraordinary resistance to ionizing radiation, ultraviolet light, and desiccation, has emerged as a pivotal model for elucidating radiation resistance mechanisms [6,7,8,9,10,11]. Its exceptional resilience is attributed to its genomic redundancy, an efficient Mn^2+^-based antioxidant system [12,13], and specialized DNA repair pathways [14,15,16,17]. Recent advancements in molecular biology and omics technologies have significantly enhanced our understanding of *D. radiodurans*, particularly through the discovery of the global regulator radiation response metalloprotease PprI, which has provided profound insights into its radiation resistance mechanisms [18,19,20,21].

PprI acts as a central regulatory hub in *D. radiodurans*, serving as a master “molecular switch” that coordinates the DNA damage response [22,23]. Through phosphorylation, PprI activates the expression of RecA [24] recombinase and the nucleic acid-binding protein PprA [25,26,27,28] while simultaneously regulating the activity of critical antioxidant enzymes, such as catalase (KatE1) [29] and superoxide dismutase (SodA). In a seminal study, the Hua Yuejin group elucidated the mechanism of the PprI-DdrO regulatory axis: PprI cleaves the C-terminal α-helix of the transcriptional repressor DdrO, leading to the derepression of DNA repair genes (e.g., recA, *pprA*) and the initiation of a comprehensive repair program [30]. Transcriptomic analyses further demonstrate that PprI governs over 200 genes under radiation stress, encompassing redox homeostasis, metabolic reprogramming, and the chaperone system [31]. Although these findings highlight the crucial role played by PprI at the transcriptional level, its global functions in post-translational modifications, protein–protein interactions, and metabolic regulation remain largely underexplored.

Despite notable progress being made in deciphering the gene regulatory functions of PprI, a critical gap remains in understanding its dynamic regulatory landscape at the proteomic level. Traditional techniques, such as two-dimensional electrophoresis, are constrained by low resolution and have identified only a few dozen differentially abundant proteins, which is insufficient to comprehensively map the PprI regulatory network [32]. Although the regulatory role of PprI at the genetic level has been extensively investigated [33], its global regulatory functions at the proteomic level remain poorly understood. In recent years, proteomic technologies have made remarkable advancements in the high-throughput quantitative analysis of protein abundance levels, modifications, and interactions, providing a powerful platform for the in-depth exploration of PprI [34].

In this study, we employed label-free quantitative proteomics to systematically compare the proteomic profiles of the *pprI* knockout strain and wild-type strain of *D. radiodurans*, identifying a series of significantly differentially abundant proteins, which are broadly involved in key biological processes such as DNA repair, antioxidant defense, and metabolic reprogramming. By integrating proteomic data with functional analysis, this study utilized advanced proteomic approaches to demonstrate that PprI deficiency triggers a systemic response, with coordinated perturbations in genome maintenance and metabolic networks despite preserved viability. These proteomic insights into PprI’s constitutive regulatory functions may inform future studies on stress adaptation mechanisms.

## 2. Materials and Methods

### 2.1. Bacterial Strains and Chemical Reagents

*D. radiodurans* (DR) was purchased from CGMCC (Beijing, China) (No. 1.633). The *pprI* knockout strain *D. radiodurans*/Δ*pprI* (Δ*pprI*) was provided by Professor Hua Yuejin, Zhejiang University, Zhejiang Province, China. The two strains were grown in TGY medium (1% tryptone, 0.5% glucose, 0.1% yeast extract) at 28 °C and 130 rpm/min until reaching the logarithmic growth phase (OD_600_ ~ 1.0) for further proteomics analysis.

For plotting the growth curves, both the DR and Δ*pprI* strains were cultured in sterilized fresh TGY liquid medium at 28 °C with shaking at 130 rpm. The absorbance at optical density (OD_600_) for each strain was measured every two hours in three biological replicates. Based on the OD_600_ values, the growth curves of both the DR and Δ*pprI* strains were plotted.

Urea, dithiothreitol (DTT), and ammonium bicarbonate were procured from Sigma-Aldrich (St. Louis, MO, USA). Trypsin was obtained from Promega (Madison, WI, USA). Formic acid and acetonitrile (ACN) were sourced from Thermo Fisher Scientific (Waltham, MA, USA). Protease inhibitors were supplied by Roche (Basel, Switzerland). The bicinchoninic acid (BCA) protein assay kit and BCA peptide assay kit were purchased from Thermo Fisher Scientific (Waltham, MA, USA).

### 2.2. Sample Preparation for Proteomics Analysis

For the proteomics analysis, quadruplicate biological replicates were prepared for each strain. The wild-type strain (DR) and the *pprI* knockout strain (Δ*pprI*) cultures were centrifuged at 10,000× *g* for 10 min at 4 °C, and the pellets were collected and washed twice with 50 mM phosphate-buffered saline (PBS). The bacterial pellets were collected and resuspended in lysis buffer (8 M urea, 2 mM EDTA, protease inhibitor mixture, 50 mM NH_4_HCO_3_). Cell lysis was performed via ultrasonic treatment (800 W, 20 kHz; 2 s on, 2 s off) in an ice-water bath for 4 min, followed by centrifugation at 15,000× *g* for 20 min at 4 °C. The supernatant was transferred to a new tube. Each sample was reduced with 10 mM of DTT for 30 min at 56 °C, alkylated with 50 mM of IAA in the dark for 30 min at room temperature, and then diluted and digested for 18 h at 37 °C by trypsin. Trypsin activity was quenched by the addition of 1% formic acid (*v*/*v*). The peptides were desalted using a C_18_ solid-phase extraction (SPE) column from Sigma-Aldrich (St. Louis, MO, USA) and dried using a vacuum centrifuge. The peptide concentrations were determined using the BCA peptide assay kit.

### 2.3. LC-MS/MS Analysis

The peptide mixture was dissolved in water containing 0.1% formic acid (FA) and analyzed using an online U3000-nano system coupled with an Orbitrap Q-Exactive HFX mass spectrometer (Thermo Fisher Scientific, Waltham, MA, USA). Peptides were separated on a 15 cm in-house C_18_ reversed-phase column (100 μm inner diameter, 1.9 μm resin) with a 130 min elution gradient. Mobile phase A consisted of 0.1% FA in water, and mobile phase B consisted of 20% water and 80% acetonitrile. The gradient program was as follows: 0% B at 0 min, 6% B at 10 min, 10% B at 18 min, 0% B at 20 min, 30% B at 102 min, 40% B at 118 min, 95% B at 118.1 min, 95% B at 125 min, 6% B at 125.1 min, and 6% B at 130 min. The flow rate was set to 300 nL/min. Data were acquired in a data-dependent mode with the *m*/*z* range for MS scans set to 300–1550 *m*/*z* and an accumulation time of 0.25 s. The top 20 most intense ions in MS1 were selected for MS/MS analysis, with a dynamic exclusion time of 20 s. The mass spectrometry proteomics data have been deposited to the ProteomeXchange Consortium via the PRIDE partner repository with the dataset identifier PXD062500. You can log in to the PRIDE website (https://www.ebi.ac.uk/pride, accessed on 2 April 2025) using the following details: Username: mail to: reviewer_pxd062500@ebi.ac.uk; Password: 8AWIm9jNBLAr.

### 2.4. Data Analysis and Bioinformatics

The RAW mass spectrometry files were processed using pFind 3.2.0 [35] and pQuant v1.0 [36] software with the *D. radiodurans* database set downloaded from UniProtKB (https://www.uniprot.org/; uniport proteome_UP000002524, (accessed on 2 October 2019)). A false discovery rate (FDR) of 0.01 was applied at both the protein and peptide levels. The search parameters included a first search peptide tolerance of 20 ppm, a main search peptide tolerance of 4.5 ppm, trypsin specificity with a maximum of two missed cleavages, carbamidomethylation (C) as a fixed modification, and oxidation (M) as a variable modification. Other parameters in pFind were set to default values.

Label-free quantification (LFQ) was performed using the built-in algorithm in pQuant, and the results were imported into Perseus software (https://maxquant.net/perseus/) for statistical analysis. Statistical significance was determined using the Benjamini–Hochberg procedure to control the false discovery rate (FDR) at 5% (adjusted *p*-value < 0.05). Proteins with |log_2_FC| > 1 (|fold change| > 2) and adjusted *p*-value < 0.05 were classified as significantly differentially abundant. Functional annotation and categorization of differentially abundant proteins were performed using DAVID 6.8 bioinformatics tools (https://david.ncifcrf.gov/, accessed on 17 March 2025), including gene ontology (GO) analysis. Clusters of Orthologous Groups (COG) analysis was conducted using ResearchEasy (https://researcheasy.cn/, accessed on 1 April 2025). Visualization of the results was accomplished using the following platforms: Microbiome Bioinformatics (http://www.bioinformatics.com.cn, accessed on 1 April 2025), ResearchEasy (https://researcheasy.cn/, accessed on 1 April 2025), and XianTao Academic (https://www.xiantaozi.com, accessed on 27 March 2025). Protein–protein interaction (PPI) networks were constructed and visualized using Cytoscape (version 3.10.3) based on interaction data retrieved from the STRING database. Pathways with *p*-value < 0.05 were considered statistically significant and included in the analysis. The Kappa consistency factor was set at 0.7.

## 3. Results

### 3.1. Growth Curves of the DR and ΔpprI Strains

The growth dynamics of the DR and Δ*pprI* strains were systematically monitored under identical culture conditions for 36 h (Figure S1). Both strains were inoculated at the same initial OD_600_ (~0.1) and exhibited distinct growth profiles. The DR strain transitioned rapidly into the logarithmic growth phase (4–20 h) and grew faster, whereas the Δ*pprI* strain displayed a significantly slower logarithmic growth. By 24 h, the DR strain entered the stationary phase, stabilizing at an OD_600_ of around 1.7. In contrast, the Δ*pprI* strain exhibited prolonged growth, with OD_600_ values continuing to increase until 34 h, reaching a final OD_600_ of around 2.0 before stabilizing.

### 3.2. Comparative Proteomic Analysis of Total Proteins in Wild-Type and pprI Knockout Strains

To assess the effects of *pprI* gene deletion on protein abundance levels in *D. radiodurans*, label-free quantitative proteomics was employed to extract and quantify total proteins from the DR and Δ*pprI* strains. Quadruplicate biological replicates were prepared for each group, and 2522 proteins were identified using pFind 3.2.0 software (Table S1).

**Figure 1:**
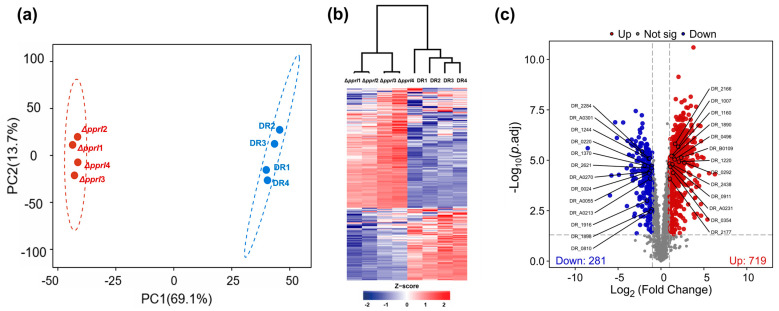
Quality assessment of proteomic data of wild-type strain and *pprI* knockout strains. (**a**) Principal component analysis (PCA) of the DR and Δ*pprI* strains. (**b**) Heatmap showing the abundance of 2522 proteins identified in the wild-type strain and the knockout strain. (**c**) Volcano plot showing proteins from the proteomic analysis of the DR and Δ*pprI* strains. Proteins with significantly differential abundance (|log_2_FC| > 1; *p*-adj < 0.05) in the blue and red dots represent lower and higher abundance of proteins in the knockout strain compared to wild-type strains. (The 2-fold threshold was chosen to prioritize proteins with substantial abundance changes, while the Benjamini–Hochberg-adjusted *p*-value < 0.05 ensured statistical rigor.) Non-significant proteins are shown in gray.

To evaluate data quality, principal component analysis (PCA) was conducted. The first two principal components, PC1 and PC2, explained 69.1% and 13.7% of the total variance, respectively, cumulatively accounting for 82.8% of the data variability. The 95% confidence interval is represented by dashed ellipses. Samples from both the wild-type and knockout groups were distributed within their respective ellipses, confirming the dataset’s statistical significance. Notably, the non-overlapping confidence ellipses between the two groups indicated statistically significant differences in the PCA space. The compact clustering of points along PC1 within each group reflects high intra-group similarity and replicate reproducibility. Furthermore, the clear separation of protein profiles in the PCA plot suggests substantial proteomic alterations due to *pprI* gene deletion (Figure 1a).

The abundance profiles of 2522 proteins identified in the DR and Δ*pprI* strains were visualized using a heatmap (Figure 1b). Columns correspond to samples, while rows represent proteins. Z-score normalization was applied, with red, blue, and gray indicating the high abundance of proteins, low abundance of proteins, and proteins with missing quantification data, respectively. The heatmap demonstrated high intra-group similarity but distinct inter-group differences. These distinct abundance patterns effectively differentiate the wild-type and knockout groups, indicating that *pprI* deletion induces widespread differentially abundant proteins. These findings highlight the pivotal regulatory role of PprI in protein abundance levels, aligning with prior studies that identify PprI as a global regulator.

To identify significantly differentially abundant proteins, a threshold of |log_2_FC| > 1 and an adjusted *p*-value (*p*-adj) of <0.05 were applied, as visualized in the volcano plot (Figure 1c). Proteins with high fold changes and significant *p*-adj values were prominently clustered in the upper left and right quadrants of the plot. A total of 1000 proteins exhibited significantly differential abundance between the wild-type and knockout strains, with a 719 higher and 281 lower abundance of proteins in the Δ*pprI* over DR strains.

### 3.3. Gene Ontology (GO) and Clusters of Orthologous Groups (COG) Analysis

#### 3.3.1. Gene Ontology Analysis

**Figure 2:**
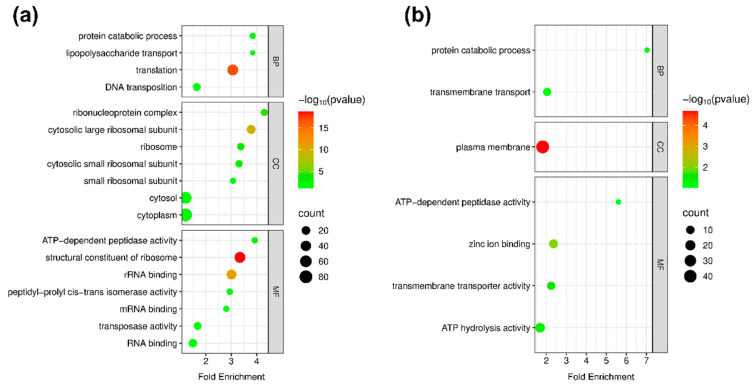
Gene ontology enrichment analysis of differentially abundant proteins. (**a**) High abundance of proteins enriched with GO annotations. (**b**) Low abundance of proteins enriched with GO annotations. The enriched terms were visualized based on their log10-transformed *p*-values and the number of associated proteins (represented by dot size).

To investigate the biological roles of differentially abundant proteins between the DR and Δ*pprI* strains, GO enrichment analysis was performed on high- and low-abundance proteins using DAVID 6.8 and the Microbiome Bioinformatics platform. Significant GO terms were identified using an EASE score threshold of 0.1 and classified into biological process (BP), cellular component (CC), and molecular function (MF) categories (Figure 2a,b).

The GO analysis revealed the critical regulatory role played by PprI in *D. radiodurans*. In the BP category, a high abundance of proteins were enriched in protein catabolic processes, lipopolysaccharide transport, and translation, suggesting PprI’s involvement in stress responses and energy metabolism through mechanisms such as protein turnover, membrane integrity preservation, and translational regulation. Conversely, a low abundance of proteins were associated with protein catabolic processes and transmembrane transport, indicating potential suppression of these pathways in the knockout strain, which may impair protein homeostasis and membrane functionality, thereby reducing radiation resistance and stress adaptation. In the CC category, a high abundance of proteins were mainly localized to the ribosome, cytosol, and cytoplasm, pointing to enhanced ribosomal composition and function in the knockout strain. In contrast, a low abundance of proteins were linked to the plasma membrane, suggesting disruption of membrane-related activities in the absence of PprI. Within the MF category, a high abundance of proteins were enriched in ATP-dependent peptidase activity, ribosomal structural constituents, and mRNA binding, highlighting PprI’s role in protein quality control, translational efficiency, and gene expression regulation. A low abundance of proteins were associated with ATP hydrolysis activity, zinc ion binding, and transmembrane transporter activity, indicating the potential inhibition of transmembrane transport and energy metabolism in the knockout strain.

#### 3.3.2. Clusters of Orthologous Groups Analysis

Based on the COG functional classification analysis, the functional distribution of up- and low-abundance proteins was systematically compared between the DR and the Δ*pprI* strain (Tables S2 and S3). This analysis highlighted the critical role played by PprI in DNA damage repair, antioxidant response, and metabolic reprogramming, underscoring its multifaceted regulatory functions.

**Figure 3:**
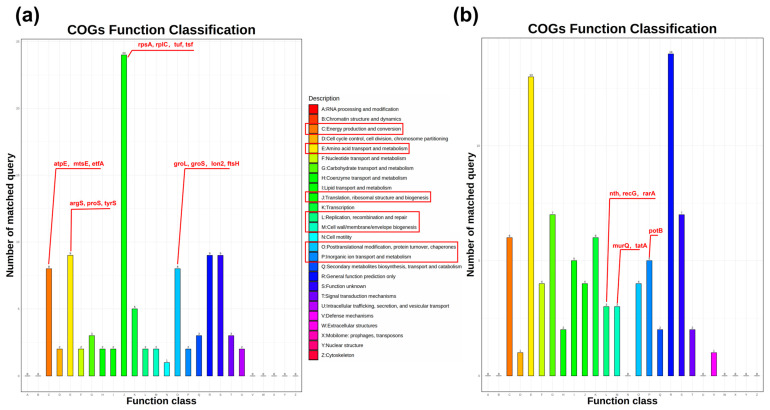
COG analysis of differentially abundant proteins. (**a**) High abundance of protein COG function annotation. (**b**) Low abundance of protein COG function annotation. The COG functional categories with more than five matched query gene names are highlighted in red boxes, and key genes associated with DNA repair, oxidative stress response, and metabolic reprogramming are annotated in red font.

Among the high abundance of proteins, significantly enriched COG categories included C (energy production and conversion), E (amino acid transport and metabolism), J (translation, ribosomal structure and biogenesis), and O (posttranslational modification, protein turnover, and chaperones). The enrichment of these functional categories suggests that the Δ*pprI* strain likely activates the compensatory pathways to enhance energy metabolism and protein synthesis in response to environmental stress while simultaneously mobilizing protein quality control systems to maintain cellular homeostasis and antioxidant defense mechanisms (Table S2, Figure 3a).

Conversely, the low abundance of proteins was predominantly enriched in COG categories M (cell wall/membrane/envelope biogenesis), P (inorganic ion transport and metabolism), and L (replication, recombination, and repair). These functional declines indicate that *pprI* deletion may impair cell wall and membrane integrity, disrupt ion homeostasis, and attenuate DNA damage repair mechanisms, collectively compromising the cellular adaptability to environmental stress and genomic stability (Table S3, Figure 3b).

### 3.4. Protein–Protein Interactions and Biological Pathway Networks

**Figure 4:**
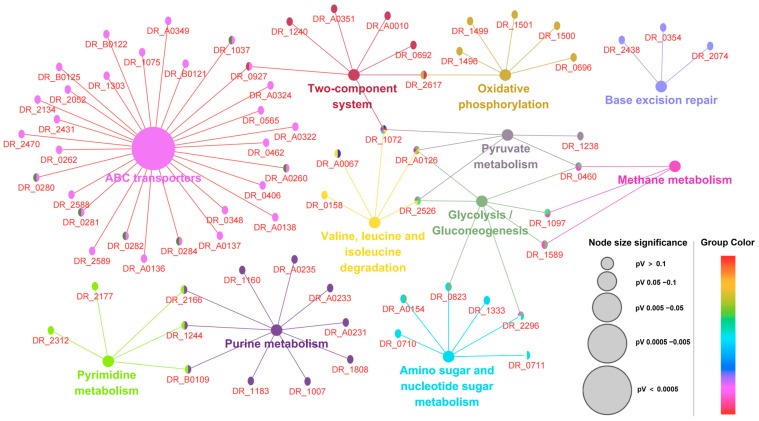
Protein–protein interaction network diagram of differentially abundant proteins based on GO pathways. Protein–protein interaction networks were constructed using the STRING database and further optimized in Cytoscape with a Kappa consistency threshold of 0.7. Different pathways are indicated by different colors, and interactions between proteins are indicated by colorful lines. The gene names in the red text indicated that the corresponding differentially abundant proteins were in the GO pathways. The protein interaction network shows 11 different connected signaling pathways.

To elucidate the molecular mechanisms underlying the extreme radiation resistance of *D. radiodurans*, we performed a systematic analysis of protein–protein interactions (PPIs) and biological pathway networks. A high-confidence PPI network was constructed using the STRING database and further optimized in Cytoscape with a Kappa consistency threshold of 0.7. This network revealed significant enrichment in 11 key GO pathways (Figure 4), including ABC transporters, two-component signaling systems, oxidative phosphorylation, base excision repair, pyrimidine and purine metabolism, amino sugar and nucleotide sugar metabolism, glycolysis/gluconeogenesis, etc. Notably, we identified PprI as a central hub coordinating DNA repair, antioxidant defense, and metabolism while facilitating cross-pathway synergy. These provide important molecular networks for characterizing the extensive regulatory networks affected by PprI and for revealing the coordinated interference between multiple stress response systems when PprI is absent.

## 4. Discussion

**Figure 5:**
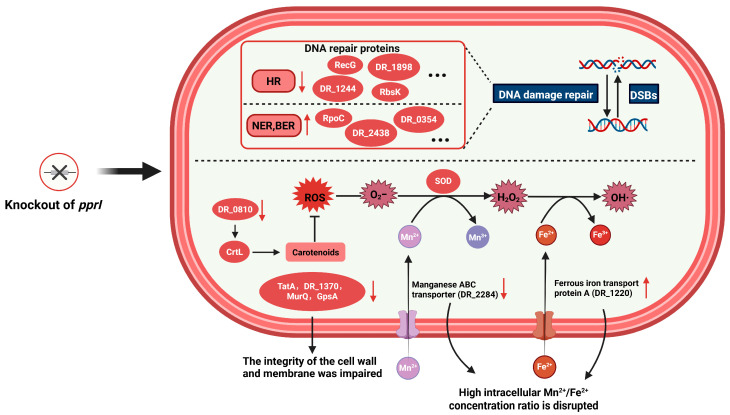
Identifying *pprI* knockout-associated changes in DNA damage repair, antioxidant defense, and metabolic reprogramming, providing a systems-level view of its regulatory influence.

*D. radiodurans*, renowned for its extraordinary resistance to radiation, serves as an exemplary model for studying cellular stress responses [18,37] and DNA repair mechanisms [1,38,39,40]. As a pivotal global regulator in *D. radiodurans*, PprI plays a central role in maintaining its extreme resistance [30,41,42]. Using label-free proteomics, we identified *pprI* knockout-associated changes in DNA damage repair, antioxidant defense, and metabolic reprogramming, providing a systems-level view of its regulatory influence [43]. These proteomic findings delineate the extensive regulatory network influenced by PprI, revealing coordinated perturbations across multiple stress response systems when PprI is absent (Figure 5).

### 4.1. Dynamic Regulation of DNA Damage Repair

Prior studies confirm PprI’s central role in *D. radiodurans’* stress response, where it coordinates DNA repair via transcriptional regulation and post-translational modifications [19,44,45]. Here, we expand this understanding by mapping PprI-dependent proteomic changes in *pprI* knockout strains. Our quantitative proteomics reveals that PprI is indispensable for maintaining genomic integrity, primarily by coordinating multiple repair systems, including homologous recombination (HR), nucleotide excision repair (NER), and base excision repair (BER) [46,47,48,49,50,51,52]. In particular, in the *pprI* knockout strain, key HR-related enzymes—ATP-dependent DNA helicase RecG (DR_1916, FC: 0.323) [53,54], AAA + ATPase domain-containing protein (DR_1898, FC: 0.476), and DNA-directed DNA polymerase (DR_1244, FC: 0.375)—were markedly low in abundance, impairing HR efficiency. Concurrently, the suppression of DNA-binding response regulator (DR_A0010, FC: 0.493) and ribokinase (RbsK, DR_A0055, FC: 0.380) suggests that PprI deletion disrupts both transcriptional control and phosphorylation-mediated signaling in DNA damage responses. Notably, compensatory mechanisms were activated: The NER-associated DNA-directed RNA polymerase subunit beta’ (RpoC, DR_0911, FC: 2.011) [55] and exodeoxyribonuclease III (DR_0354, FC: 2.669) were highly abundant, potentially offsetting HR defects by enhancing NER capacity. Similarly, endonuclease III (DR_2438, FC: 2.513), a BER core component, exhibited significant induction, underscoring a hierarchical yet flexible repair network under PprI regulation (Table 1, Figure 5).

### 4.2. Molecular Basis of Antioxidant Defense

PprI coordinates a multifaceted antioxidant defense system, primarily by modulating Mn/Fe homeostasis and activating redox-sensitive enzymes [56,57]. Quantitative proteomics demonstrated that PprI deletion perturbed Mn/Fe equilibrium, characterized by the high abundance of ferrous iron transport protein A (DR_1220, FC: 4.245) and low abundance of manganese ABC transporter (DR_2284, FC: 0.217). This dyshomeostasis presumably elevates intracellular Fe^2+^ pools, exacerbating Fenton reaction-driven oxidative damage [58]. Additionally, key antioxidant components were compromised: cytochrome-c peroxidase (DR_A0301, FC: 0.400) and the dehydrogenases associated with carotenoid biosynthesis (DR_0810, FC: 0.436) [59,60,61] were significantly low in abundance, impairing ROS scavenging capacity. Conversely, the compensatory induction of ferredoxin/ferredoxin--NADP reductase (DR_0496, FC: 3.009) and oxidoreductase (DR_1890, DR_A0231, FC: 2.653) [62] suggests an adaptive response to mitigate oxidative stress through alternative redox pathways (Table 2, Figure 5).

### 4.3. Metabolic Reprogramming

The Δ*pprI* strain underwent extensive metabolic rewiring, characterized by dysregulated nucleotide metabolism and compromised cell envelope architecture. De novo purine biosynthesis was severely attenuated due to the downregulation of amidophosphoribosyltransferase (PurF, DR_0220, FC: 0.469) and N5-carboxyaminoimidazole ribonucleotide synthase (PurK, DR_0024, FC: 0.387) [63], along with adenine deaminase (Ade, DR_A0270, FC: 0.417), which further restricted purine salvage efficiency. In contrast, the pyrimidine salvage pathway was robustly activated, as demonstrated by the induction of cytidine deaminase (DR_2177, FC: 2.044) and uridine phosphorylase (DR_2166, FC: 3.123), suggesting a metabolic shift toward nucleotide recycling to sustain genomic stability. Strikingly, ribonucleotide–diphosphate reductase (DR_B0109, FC: 5.144), critical for de novo dNTP synthesis, was hyperactivated, potentially fueling DNA repair under replication stress. The concurrent upregulation of uricase (DR_1160, FC: 2.339) [64] and MutT/nudix family protein (DR_1007, FC: 4.027) [65] implied enhanced nucleotide catabolism linked to oxidative stress adaptation. Structural integrity was further compromised by the coordinated suppression of (i) N-acetylmuramic acid 6-phosphate etherase (MurQ, DR_A0213, FC: 0.420); (ii) Sec-independent protein translocase protein TatA (DR_0292, FC: 0.419); (iii) Glycerol-3-phosphate dehydrogenase [NAD(P)+] (GpsA, DR_2621, FC: 0.469); and (iv) outer membrane lipoprotein-sorting protein (DR_1370, FC: 0.266), collectively impairing membrane homeostasis (Table 3, Figure 5).

### 4.4. Limitations of This Present Study

While this study systematically delineates PprI’s proteome-wide regulatory network in *D. radiodurans*, several limitations warrant consideration. Firstly, the label-free quantitative proteomics approach, while robust for profiling canonical ORF-encoded proteins, lacks resolution for proteoforms (including post-translational modifications [PTMs], e.g., phosphorylation or proteolytic cleavage) and alternative isoforms, potentially overlooking critical regulatory events. Secondly, despite high-resolution mass spectrometry, low-abundance proteins (e.g., transcription factors) may evade detection, omitting minor but functionally significant nodes in the PprI network. Thirdly, our analysis was conducted under unstressed conditions, limiting extrapolation to PprI’s dynamic role during radiation-induced stress responses; the observed proteomic shifts may reflect constitutive rather than stress-activated regulation at the proteoform level. Lastly, the absence of systematic transcriptomic–proteomic integration precludes the direct correlation of transcriptional regulation with observed proteomic changes. These limitations highlight the need for future integrative studies employing PTM-enriched proteoform analysis, multi-omics approaches, and targeted functional experiments to fully dissect PprI’s hierarchical control over stress adaptation.

## 5. Conclusions

*D. radiodurans* stands as a paragon of extremophile biology, renowned for its extraordinary resistance to ionizing radiation, desiccation, and oxidative stress. This resilience is attributed to a multifaceted defense system encompassing efficient DNA repair machinery, robust antioxidant networks, and unique proteomic adaptations. Central to this system is the radiation-induced metalloprotease PprI, a master regulator previously shown to orchestrate stress responses. However, while genetic and transcriptomic studies have illuminated PprI’s role in acute stress survival, its global proteomic regulatory landscape—particularly under basal conditions—remains poorly characterized. Therefore, protein mapping based on high-throughput proteomics for the in-depth study of protein abundance and interactions in the absence of PprI becomes more important.

In this comprehensive study, we employed label-free quantitative proteomics coupled with high-resolution mass spectrometry to systematically characterize the proteomic landscape of *D. radiodurans* wild-type and *pprI* knockout strains, unraveling coordinated perturbations across multiple stress response systems when PprI is absent. By comparing the proteomic profiles of DR and Δ*pprI* strains, we identified 1000 significantly differentially abundant proteins (719 with high abundance and 281 with low abundance) and found that the differentially abundant proteins were concentrated in DNA damage repair, antioxidant defense, and metabolic reprogramming, providing a systems-level view of its regulatory influence. The key findings revealed that PprI deficiency disrupts homologous recombination (HR) repair, while the compensatory activation of nucleotide excision repair (NER) and base excision repair (BER) pathways ensures genome integrity. Concurrently, impaired Mn/Fe homeostasis and reduced carotenoid biosynthesis exacerbate oxidative stress, mitigated partially by the adaptive upregulation of ferredoxin/ferredoxin--NADP reductase and oxidoreductase. Metabolic perturbations include dysregulated purine synthesis and compromised cell envelope integrity, counterbalanced by activated pyrimidine salvage and enhanced dNTP production. These findings collectively illustrate PprI’s foundational role in maintaining genomic and metabolic stability in *D. radiodurans*, offering a proteomic blueprint that bridges its known stress-responsive functions with constitutive regulatory mechanisms. While this study identifies compensatory adaptations that may sustain viability under PprI deficiency, the absence of stress-induction experiments limits direct inferences regarding radiation resistance. Future work integrating targeted mutagenesis and multi-omics approaches under ionizing radiation will be critical to dissecting PprI’s dynamic role in extremophile survival.

## Figures and Tables

**Table 1:** Differentially abundant proteins associated with DNA damage repair mechanisms.

Accession ID	Protein Name	Gene Name	Fold Change	COG Category	Protein Score
Q9RXH8	RNA helicase	DR_0335	3.217	LKJ	875.0
**Q9RXF9**	**Exodeoxyribonuclease III**	**DR_0354**	**2.669**	**L**	**577.0**
Q9RZ89	DNA-binding protein HU	DR_A0065	2.642	L	186.0
**Q9RRQ0**	**Endonuclease III**	**DR_2438**	**2.513**	**L**	**540.0**
Q9RSJ6	DNA-directed RNA polymerase subunit alpha	DR_2128	2.187	K	662.0
Q9RYM2	ATP-dependent zinc metalloprotease FtsH	DR_A0290	2.141	O	1270.0
Q9RXG4	Lon protease	DR_0349	2.108	O	1573.0
**Q9RVW0**	**DNA-directed RNA polymerase subunit beta’**	**DR_0911**	**2.011**	**K**	**3033.0**
Q9RSC5	Orotidine-5′-phosphate decarboxylase	DR_2200	0.498	F	520.0
Q9RW54	GntR family transcriptional regulator	DR_0815	0.494	K	508.0
Q9RZD9	DNA-binding response regulator	DR_A0010	0.493	T	396.0
**Q9RT67**	**AAA + ATPase domain-containing protein**	**DR_1898**	**0.476**	**L**	**843.0**
Q9RV39	A-adding tRNA nucleotidyltransferase	DR_1191	0.385	J	859.0
**Q9RZ99**	**Ribokinase**	**DR_A0055**	**0.380**	**H**	**573.0**
**Q9RUY6**	**DNA-directed DNA polymerase**	**DR_1244**	**0.375**	**L**	**575.0**
**Q9RT50**	**ATP-dependent DNA helicase RecG**	**DR_1916**	**0.323**	**LK**	**1517.0**
Q9RSQ0	Putative 3-methyladenine DNA glycosylase	DR_2074	0.183	L	392.0

**Table 2:** Differentially abundant proteins associated with antioxidant defense mechanisms.

Accession ID	Protein Name	Gene Name	Fold Change	COG Category	Protein Score
**Q9RV10**	**Ferrous iron transport protein A**	**DR_1220**	**4.245**	**P**	**152.0**
**Q9RX19**	**Ferredoxin/Ferredoxin--NADP reductase**	**DR_0496**	**3.009**	**ER**	**939.0**
**Q9RT75**	**Oxidoreductase**	**DR_1890**	**2.653**	**C**	**650.0**
**Q9RYS7**	**Oxidoreductase**	**DR_A0231**	**2.122**	**C**	**1455.0**
Q9RVS5	Isochorismatase family protein	DR_0947	0.489	Q	228.0
**Q9RW59**	**Dehydrogenase**	**DR_0810**	**0.436**	**Q**	**901.0**
Q9RU93	NADH-quinone oxidoreductase	DR_1499	0.422	C	1398.0
**Q9RYL1**	**Cytochrome-c peroxidase**	**DR_A0301**	**0.400**	**P**	**687.0**
**Q9RS43**	**Manganese ABC transporter, ATP-binding protein**	**DR_2284**	**0.217**	**P**	**482.0**

**Table 3:** Differentially abundant proteins associated with metabolic reprogramming.

Accession ID	Protein Name	Gene Name	Fold Change	COG Category	Protein Score
Q9RW75	[LysW]-aminoadipate semialdehyde transaminase	DR_0794	5.494	E	835.0
**Q9RZL6**	**Ribonucleotide–diphosphate reductase subunit beta**	**DR_B0109**	**5.144**	**F**	**603.0**
**Q9RVM0**	**MutT/nudix family protein**	**DR_1007**	**4.027**	**L**	**324.0**
Q9RU24	ABC transporter substrate-binding protein	DR_1571	3.378	E	1194.0
Q9RSW3	Aspartate-semialdehyde dehydrogenase	DR_2008	3.351	E	660.0
Q9RWQ9	Chaperonin GroEL	DR_0607	3.219	O	1016.0
Q9RUB7	Phage shock protein A homolog	DR_1473	3.137	KT	403.0
**Q9RSF8**	**Uridine phosphorylase**	**DR_2166**	**3.123**	**F**	**612.0**
Q9RW01	Bifunctional purine biosynthesis protein PurH	DR_0868	3.068	F	1002.0
Q9RUW4	Proline--tRNA ligase	DR_1266	3.004	J	1021.0
Q9RRC4	Arginine--tRNA ligase	DR_2568	2.867	J	1194.0
Q9RXK2	Large ribosomal subunit protein uL3	DR_0311	2.733	J	419.0
Q9RY06	Valine--tRNA ligase	DR_0148	2.596	J	1840.0
Q9RUP2	Phosphoglycerate kinase	DR_1342	2.563	G	805.0
Q9RR70	Fumarate hydratase class II	DR_2627	2.552	C	919.0
Q9RSL8	Fe-S cluster assembly protein SufB	DR_2106	2.525	O	942.0
Q9RY67	2-oxoglutarate dehydrogenase complex dihydrolipoyllysine-residue Succinyltransferase(odhB)	DR_0083	2.489	C	783.0
Q9RYB2	Serine hydroxymethyltransferase	DR_0038	2.462	E	810.0
O32507	Succinate-semialdehyde dehydrogenase [NADP(+)]	DR_A0343	2.455	C	936.0
Q9RUF5	Phosphoribosylamine--glycine ligase	DR_1431	2.396	F	811.0
**Q9RV70**	**Uricase**	**DR_1160**	**2.339**	**Q**	**617.0**
Q9RWB2	Citrate synthase	DR_0757	2.322	C	746.0
Q9RVQ2	Electron transfer flavoprotein, alpha subunit	DR_0970	2.307	C	597.0
Q9RRA0	Magnesium protoporphyrin chelatase	DR_2594	2.272	H	949.0
Q9RWH2	V-type ATP synthase, K subunit	DR_0696	2.268	C	170.0
Q9RS27	Alanine--tRNA ligase	DR_2300	2.231	J	1748.0
Q9RR63	Tyrosine--tRNA ligase	DR_2634	2.070	J	807.0
Q9R342	Elongation factor Tu	DR_0309	2.064	J	797.0
**Q9RSE7**	**Cytidine deaminase**	**DR_2177**	**2.044**	**F**	**317.0**
Q9RVK7	ATP-dependent zinc metalloprotease FtsH	DR_1020	0.498	O	1201.0
Q9RYY2	Hydroxymethylpyrimidine kinase	DR_A0171	0.491	H	486.0
**Q9RXT6**	**Amidophosphoribosyltransferase**	**DR_0220**	**0.469**	**F**	**959.0**
**Q9RR76**	**Glycerol-3-phosphate dehydrogenase [NAD(P)+]**	**DR_2621**	**0.469**	**I**	**625.0**
Q9RVF3	Cell wall synthesis protein	DR_1076	0.454	M	813.0
**Q9RYU5**	**N-acetylmuramic acid 6-phosphate etherase**	**DR_A0213**	**0.420**	**S**	**565.0**
**Q9RXL8**	**Sec-independent protein translocase protein TatA**	**DR_0292**	**0.419**	**U**	**211.0**
**Q9RYP0**	**Adenine deaminase**	**DR_A0270**	**0.417**	**F**	**1031.0**
**Q9RYC6**	**N5-carboxyaminoimidazole ribonucleotide synthase**	**DR_0024**	**0.387**	**F**	**727.0**
Q9RVJ1	Branched-chain amino acid ABC transporter, permease protein	DR_1037	0.361	E	655.0
Q9RX55	Acetate--CoA ligase	DR_0460	0.360	I	1301.0
**Q9RUL4**	**Outer membrane lipoprotein-sorting protein**	**DR_1370**	**0.266**	**M**	**414.0**

## Data Availability

The mass spectrometry proteomics data have been deposited to the ProteomeXchange Consortium via the PRIDE partner repository with the dataset identifier PXD062500.

## Supplementary Materials

The following supporting information can be downloaded at: https://www.mdpi.com/article/10.3390/proteomes13020019/s1, Figure S1: Growth curves of the wild-type strain (DR) and the *pprI*-knockout strain (Δ*pprI*).; Table S1: Differentially Abundant Proteins (DAPs).; Table S2: High abundance of proteins’ COG analysis.; Table S3: Low abundance of proteins’ COG analysis.
