# Supplementary figures and images for: Unraveling the Central Role of Global Regulator PprI in Deinococcus radiodurans Through Label-Free Quantitative Proteomics
Source: Proteomes. 2025 May 23;13(2):19. doi: 10.3390/proteomes13020019 (PMC12197293; doi:10.3390/proteomes13020019)

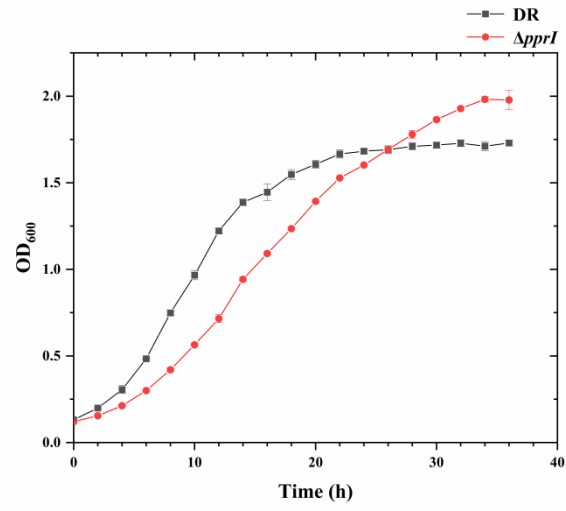

**Figure S1.** Growth curves of the wild-type strain (DR) and the *pprI*-knockout strain ( $\Delta pprI$ ).

Supplement: Supplementary file 1 [file proteomes-13-00019-s001.zip › Figure S1.pdf]
